# Intérêt de la TEP-TDM dans le cancer broncho-pulmonaire primitif non à petite cellule

**DOI:** 10.11604/pamj.2017.28.289.13130

**Published:** 2017-12-04

**Authors:** Fdil Soumia, Achachi Leila, Raoufi Mohamed, Herrak Laila, Elftouh Mustapha

**Affiliations:** 1 Service de Pneumologie, Faculté de Médecine et de Pharmacie, Université Mohammed V, CHU Ibn Sina, Rabat, Maroc

**Keywords:** TEP-TDM, CBNPC, restaging, PET-CT, non-small cell bronchopulmonary cancer, restaging

## Abstract

Le cancer broncho-pulmonaire représente un véritable problème de santé publique. L'imagerie morphologique tient une place primordiale dans le diagnostic, le bilan d'extension et l'évaluation post-thérapeutique, mais connaît certaines limites. D'apparition plus récente, l'imagerie métabolique permet d'améliorer les performances globales de l'imagerie de façon significative. Afin d'évaluer le rôle de la TEP-TDM au FDG dans le staging et le restaging du cancer broncho-pulmonaire non à petites cellules, nous avons mené une étude rétrospective, descriptive et analytique à l'Hôpital Ibn Sina et à l'Hôpital Militaire d'Instruction Mohammed V de Rabat pendant une durée de 18 mois, entre Septembre 2014 et Février 2016. Le bilan d'extension initial a relevé une grande majorité des stades localement avancés et métastatiques: stade IV (40%), stade IIIB (36%), stade IIIA (16%), stade II (8%). La TEP-TDM a permis de retrouver de nouvelles localisations non objectivées initialement à la TDM dans 24 cas: 15 nouvelles localisations ganglionnaires, 8 nouvelles localisations surrénaliennes et 6 localisations osseuses. La TEP-TDM a ainsi permis de modifier le stade dans 60% des cas, un up staging chez 23 patients (46%) et un down staging chez 7 patients (14%). Le stade initial est resté inchangé chez 40% des patients. Notre étude confirme les données de la littérature concernant la supériorité de la TEP-TDM en comparaison avec la TDM seule dans l'optimisation de la prise en charge du CBNPC, notamment dans le bilan d'extension locorégional et à distance.

## Abstract (en)

Bronchopulmonary cancer is a real public health problem. Morphological imaging plays a central role in its diagnosis, staging as well as post-therapeutic assessment but it has some limitations. Metabolic imaging is a more recent technique which allows to significantly improve the overall imagery performance. We conducted a retrospective, descriptive and analytical study at the Ibn Sina Hospital and at the Military Hospital of instruction Mohammed V in Rabat over a period of 18 months, between September 2014 and February 2016, in order to evaluate the role of Fluorodeoxyglucose-PET/CT in the staging and restaging of non-small cell bronchopulmonary cancer. Initial staging showed a vast majority of locally advanced and metastatic stages: stage IV (40%), Stage IIIB (36%), Stage IIIA (16%), Stage II (8%). PET-CT allowed to detect new sites which were not initially seen on CT scan in 24 cases: 15 new ganglion sites, 8 new adrenal sites and 6 sites of bone lesions. PET/CT allowed to modify initial tumor stage in 60% of cases: upstaging in 23 patients (46%) and downstaging in 7 patients(14%). The initial stage remained unchanged in 40% of patients. Our study confirms the data from the literature concerning the superiority of PET-CT in comparison with CT scan, but only in the optimization of the non-small cell bronchopulmonary cancer management, in particular in locoregional and distant staging.

## Introduction

Le cancer broncho-pulmonaire (CBP) est le plus fréquent dans le monde depuis plusieurs décennies et constitue le premier cancer chez l'homme. En raison de son caractère généralement agressif qui peut rapidement engendrer des métastases, le CBP est souvent diagnostiqué à un stade avancé où les stades III et IV représentent près de 80% des cas [1]. La tomographie par émission de positons couplée à la tomodensitométrie (TEP-TDM) au 18 F-fluoro-déoxy-glucose (18F-FDG) représente actuellement l'examen d'imagerie le mieux adapté pour évaluer l'extension initiale de ce type de cancer et peut modifier la prise en charge du cancer, que ce soit au niveau du staging ou du restaging et surtout de l'appréciation de l'efficacité thérapeutique. L'objectif de notre étude est de démontrer l'intérêt de la TEP-TDM dans l'optimisation de la prise en charge des cancers broncho-pulmonaires non à petites cellules par rapport au scanner thoracique, notamment dans le bilan d'extension locorégional et à distance de ces cancers.

## Méthodes

Il s'agit d'une étude rétrospective descriptive et analytique, menée au sein des services de Pneumologie de l'Hôpital Ibn Sina et de l'Hôpital Militaire d'Instruction Mohammed V de Rabat colligeant 50 cas durant une période de 18 mois, de septembre 2014 à Février 2016 et ayant recruté des patients présentant un cancer broncho-pulmonaire primitif non à petites cellules, confirmé histologiquement, ayant bénéficié d'un bilan d'extension initial et ayant fait l'objet d'une réunion de concertation pluridisciplinaire (RCP) d'oncologie thoracique. On a exclu de cette étude les cancers broncho-pulmonaires à petites cellules, ainsi que les cancers broncho-pulmonaires secondaires. Le 18-fluoro-2-désoxy-D-glucose (FDG) est injecté chez nos patients à la dose de 4 à 5 MBq/kg par voie intraveineuse directe par la tubulure d'une perfusion continue de 500ml de sérum salé. Chaque patient doit être au repos et à jeun depuis au moins 6 heures. Seule la consommation d'eau est autorisée à fin de réduire la fixation myocardique et musculaire du glucose et d'éviter ainsi une compétition entre le glucose et le FDG au niveau des transporteurs membranaires cellulaires. L'exploration est menée de la voûte crânienne jusqu'à mi-cuisse. Les paramètres d'acquisition sont: TEP-FDG 3min/pas, couplés à la TDM (120kV), permettant des coupes de 3.75mm d'acquisition volumique. Toutes les masses tumorales et leurs métastase ont bénéficié d'une quantification hypermétabolique par l'indice SUV «Standard ized Up take Value».

## Résultats

Parmi 212 patients hospitalisés pour cancer broncho-pulmonaire non à petites cellules pendant une période de 18 mois, entre septembre 2014 et février 2016, 50 patients ont bénéficié d'une TEP-TDM soit 23,5%. L'âge moyen de nos patients était de 64.5 ± 14,5 ans, avec des extrêmes allant de 52 à 81 ans. Sur les 50 patients, 92% étaient de sexe masculin, soit un sexe ratio de 11,5. Le tabagisme était retrouvé chez 45 patients soit 90% des cas, tous de sexe masculin, avec une moyenne de 48 paquets années. La TDM thoracique réalisée chez tous nos patients mettait en évidence un processus tumoral pulmonaire, proximal dans 32% des cas et périphérique dans 60% des cas. Par ailleurs, elle objectivait Des adénopathies hilaires homolatérales (N1) chez 4 patients. Des adénopathies sous-carnaires (N2) chez 8 patients. Des adénopathies controlatérales ou sus-claviculaires (N3) chez 2 patients. Une atélectasie dans 10% des cas. Une pleurésie dans 20% des cas, la biopsie bronchique a permis de poser le diagnostic chez 28% des cas et la biopsie pulmonaire écho-guidée et scanno-guidée dans 62% des cas, dans les autres cas, la biopsie pleurale et la biopsie ganglionnaire ont pu confirmer le diagnostic dans 6% et 4% des cas respectivement. Dans notre série, il s'agissait de 32 cas d'adénocarcinomes (ADK) soit un taux de 64% et 18 cas de carcinomes épidermoïdes soit 36% des cas. Le bilan d'extension comportait systématiquement un examen clinique complet. Les examens para-cliniques étaient demandés en fonction des signes d'appel, de la disponibilité des explorations et des moyens du patient. L'échographie abdominale était réalisée chez 32 patients (64%), révélant des nodules hépatiques suspects dans 5 cas et des adénopathies sous diaphragmatiques dans 9 cas. La TDM thoraco-abdomino-pelvienne (TAP) a mis en évidence des localisations hépatiques dans 7 cas, surrénaliennes dans 11 cas et osseuses dans 8 cas. La TDM cérébrale, quant à elle, a objectivé des métastases cérébrales dans 3 cas. La scintigraphie osseuse a été réalisée chez 4 patients, révélant des localisations osseuses secondaires dans les quatre cas.

**Figure 1:**
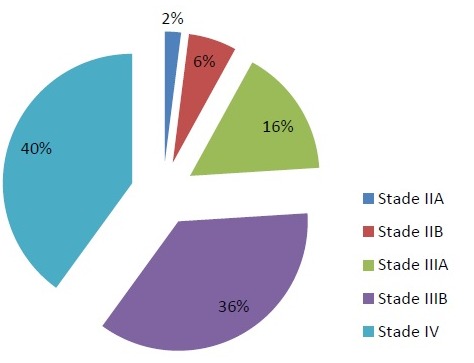
Répartition des patients en fonction des stades

**Figure 2:**
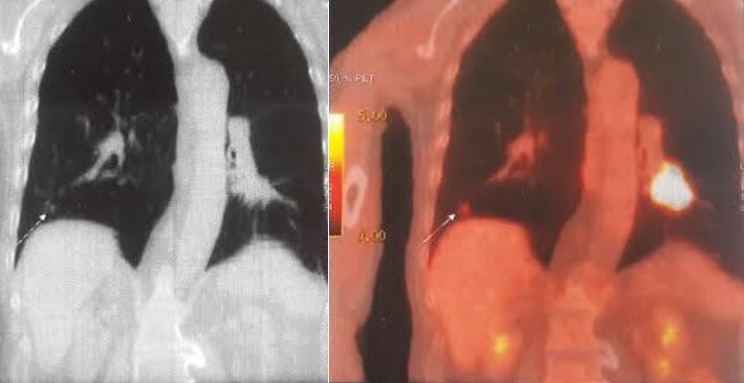
Image de fusion en TEP-TDM en coupe frontale montrant une masse pulmonaire gauche et un nodule pulmonaire controlatéral (flèche)

**Figure 3:**
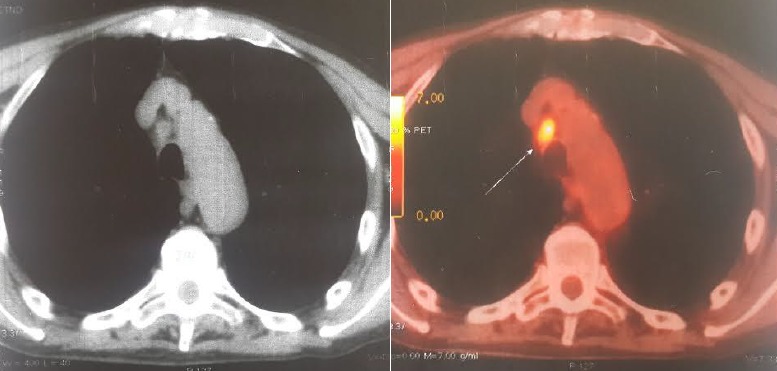
Image de fusion TEP-TDM en coupe axiale montrant une hyperfixation ganglionnaire médiastinale (flèche)

**Figure 4:**
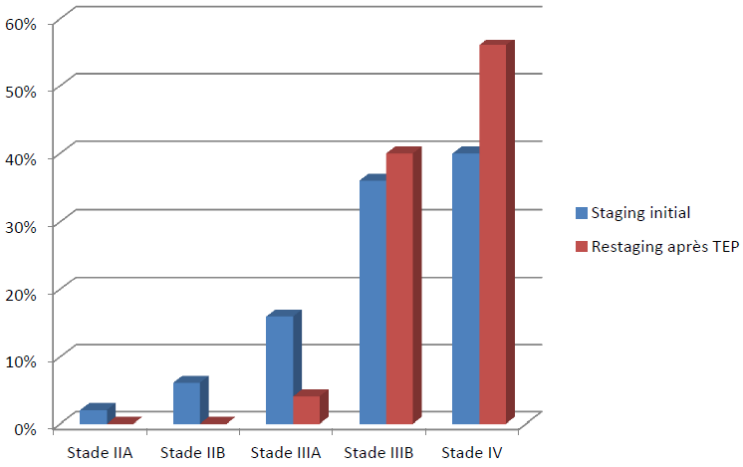
Restaging des patients après TEP-TDM au FDG

Dans la majorité des cas, les CBNPC de notre série étaient diagnostiqués à des stades III et IV, avec 2% des cas diagnostiqué au stade IIA, 6% au stade IIB, 16% au stade IIIA, 36% au stade IIIB, 40% au stade IV (Figure 1). La TEP au FDG n'a été indiquée en première intention dans le bilan d'extension chez aucun de nos patients. Elle a été indiquée dans le cadre d'un bilan de pré-opérabilité dans la majorité des cas (96%) et afin de préciser la masse à irradier dans 2 cas (Figure 2 et Figure 3). Au total, la TEP-TDM a permis de retrouver de nouvelles localisations non objectivées initialement à la TDM dans 24 cas: 15 nouvelles localisations ganglionnaires, 8 nouvelles localisations surrénaliennes, toutes unilatérales et 6 nouvelles localisations osseuses (Tableau 1). Le SUV max moyen des masses tumorales pulmonaires était de 10,8 (extrêmes variant de 3,8 à 21,8). Six patients ont bénéficié d'une médiastinoscopie pour confirmation histologique des résultats de la TEP, dont deux cas étaient faussement positifs: l'un revenant en faveur d'une anthracosilicose et le second en faveur d'une adénite réactionnelle. Un seul faux-négatif a été relevé dans notre série, correspondant à une tumeur bronchiolo-alvéolaire ne fixant pas le FDG. Le délai entre le moment du diagnostic histologique et la réalisation de la TEP-TDM était en moyenne de 22 jours et a permis de modifier le stade dans 60% des cas, un up staging chez 23 patients (soit 46% des cas) et un down staging chez 7 patients (14%). Le stade initial est resté inchangé chez 40% des patients, bien que la TEP/TDM a pu démasquer de nouvelles adénopathies hypermétaboliques appartenant aux mêmes territoires décrits sur la TDM diagnostique initiale (Figure 4). Les décisions thérapeutiques chez tous nos patients ont été prises en réunion de concertation pluridisciplinaire (pneumologues, oncologues, chirurgiens thoraciques, médecins nucléaires, radiologues, anatomopathologistes). L'indication d'un traitement associant une radiothérapie et une chimiothérapie a été retenue dans 8 cas soit 16% des cas (stade IIIB), tandis qu'une chimiothérapie exclusive a été indiquée chez 39 patients soit 78% des cas et aucun des deux patients du stade IIIA n'a pu être opéré, à cause d'une altération importante de l'état général.

**Tableau 1:** Résultats des localisations secondaires sur la TEP-TDM comparativement à la TDM initiale

	TDM TAP initiale	TEP-TDM au FDG	SUV max moyen
Localisations ganglionnaires	14	29	5,9 ± 1,5
Localisations surrénaliennes	11	19	7,3 ± 1,8
Localisations osseuses	8	14	4,8 ± 2,4

## Discussion

Le CBP est le cancer le plus fréquent dans le monde depuis plusieurs décennies. Le nombre de nouveaux cas a été estimé à 1,8 millions en 2012 (12,9% du total) dont 58% dans les régions les moins développées [1, 2]. Il représente la 1ère cause de décès par cancer. Il est classé parmi les cancers de mauvais pronostic, avec des taux de survie à 5 ans parmi les plus faibles (14% tous stades confondus) [3, 4]. Le type le plus fréquent est le cancer du poumon non à petites cellule [5, 6]. L'adénocarcinome est le sous-type le plus commun et son incidence a augmenté dans la dernière décade. L'Organisation Mondiale de la Santé (OMS) a récemment révisé les classifications histologiques de cancer du poumon, en intégrant plusieurs nouvelles lésions pré-invasives dans la classification d'adénocarcinome. Cette tendance actuelle vers l'augmentation du nombre des adénocarcinomes est justifiée par certains auteurs par le changement des habitudes toxiques, avec l'usage croissant des cigarettes à filtre et du tabac blond induisant une inhalation plus profonde et une distribution plus périphérique. Dans notre série, l'adénocarcinome est le type histologique prédominant avec une incidence de 64%, ce qui est supérieur aux résultats rapportés par Kheloui et al. Virally et al ainsi qu'aux données du RCR (Tableau 2) [4]. La TEP-TDM au 18F-FDG est un examen primordial dans le CBNPC, elle intervient à tous les stades de la prise en charge: diagnostic initial, bilan d'extension ganglionnaire locorégional et bilan d'extension à distance, optimisation des champs de radiothérapie, évaluation de la réponse tumorale à la thérapeutique, détection de la maladie résiduelle ou d'une récidive éventuelle. Néanmoins, le risque de faux négatif et de faux positifs n'est pas négligeable. Cet examen joue aussi un rôle déterminant dans le bilan d'extension du CBP, tant ganglionnaire que métastatique, compte tenu des limites de l'imagerie morphologique d'une part et du caractère invasif de la médiastinoscopie d'autre part. Plusieurs études montrent l'intérêt de la TEP-TDM dans le diagnostic des nodules pulmonaires suspects de malignité dont le diamètre dépasse 10mm, avec une sensibilité et une spécificité de l'ordre de 96,8% et 77% respectivement [6-8].

**Tableau 2:** Comparaison des types histologiques avec d’autres séries

	Kheloui et al	RCR	Virally et al	Notre série
**ADK**	55,5%	50%	46%	64%
**CE**	40%	43%	32%	36%

La TEP au FDG permet de détecter la récidive après chirurgie, chimiothérapie ou radiothérapie de manière plus précoce et plus fiable que la TDM. En effet, Gambhir et al. [9] ont démontré un écart de sensibilité de 16% entre TEP (98%) et TDM (72%), mais avec une spécificité comparable (respectivement 92% et 95%).Dans une série de 62 patients opérés et suspects de récidive, la TEP a correctement identifié 51 des 56 cas de récidives et 16 des 18 cas de rémission [10, 11]. Dans cette indication, comme lors du bilan initial, la TEP couplée à la TDM s'avère supérieure non seulement à la TDM, mais aussi à la TEP seule. La littérature est riche en publications internationales précisant le rôle incontournable de la TEP dans le bilan d'extension locorégionale. Les deux plus importantes séries, celles de Vansteenkiste et de Pieter man, chiffrent l'intérêt de la TEP: une sensibilité de 91-93%, une spécificité de 86-95%, une VPP de 74-93%, une VPN de 95% et une exactitude diagnostique de 87-94%. Toutes les études sont unanimes sur la supériorité de la TEP en comparaison avec la TDM seule (Tableau 3). La méta-analyse de Fisher est concordante avec une sensibilité de 83% et une spécificité de 96% diagnostique de 87-94%. Néanmoins la fusion TEP et TDM est plus performante que la TEP seule [12]. Cette complémentarité apporte un gain de sensibilité et de spécificité [13]. Dans l'étude de Pérotin et al, la TEP-TDM a permis une classification N pertinente dans 93% des cas versus 73% pour la TDM seule, induisant un changement thérapeutique dans 16% des cas [14, 15]. Gambhir et al [10] ont également démontré pour la détection des stades N2 et N3, sur un total de 4005 patients, un écart de sensibilité de 19% en faveur de la TEP par rapport à la TDM (83% versus 64%) et un écart de spécificité de 17% (91% versus74%). La TEP au FDG permet de mettre en évidence des métastases à distance non suspectées sur les examens conventionnels chez au moins 10% des patients. Elle modifie le stade M dans presque 20% des cas, en l'augmentant dans la plupart des cas, ce qui permet d'éviter des thoracotomies inutiles. Une modification de la stratégie thérapeutique prévue avant la réalisation de la TEP-TDM survient dans 18 à 62% des cas selon différentes études de la littérature [16]. L'influence de la technique TEP sur la connaissance de l'extension est corrélée au stade retenu avant sa réalisation. En effet, plus le stade initial est élevé, plus la probabilité de mettre en évidence une localisation à distance augmente. Mac Manus et al. [17] ont ainsi rapporté 3 localisations distantes pour 39 stades I (7,7%), 5 pour 28 stades II (18%) et 24 pour 100 stades III (24%). Par contre, la TEP au FDG ne peut déceler avec une sensibilité suffisante les localisations secondaires cérébrales, du fait de la forte consommation physiologique cérébrale de glucose, sa sensibilité est nettement inférieure à celle de l'IRM cérébrale.

**Tableau 3:** Comparaison entre TDM thoracique injectée et TEP-FDG dans la stadification ganglionnaire des CBNPC

	Sensibilité	Spécificité
**Scanner injecté**	57–61 %	78–82 %
**TEP-FDG**	53–83 %	89–92 %

C'est pour la détection des localisations surrénaliennes que la TEP est la plus performante. En effet, les anomalies surrénaliennes suspectées en TDM peuvent être caractérisées grâce à la TEP au FDG. Dans la série française de Perrotin et al, la fréquence des anomalies surrénaliennes était de 28%, la TEP avait une sensibilité de 88%, une spécificité de 100% et une VPN de 96% [16]. Dans notre série, la TEP-TDM a objectivé 8 nouvelles localisations surrénaliennes, toutes unilatérales, et dont le métabolisme intense (SUV max moyen = 7,3 ± 1,8) était en faveur d'une origine secondaire. De ce fait, aucune confrontation histologique n'a été effectuée chez nos patients. Celle-ci est recommandée en cas de fixations surrénaliennes au FDG modérées, uni ou bilatérales. La détection des métastases osseuses est également excellente par TEP, avec une précision élevée de l'ordre de 96%, nettement supérieure aux performances de la scintigraphie osseuse dont l'intérêt est très limité une fois que la TEP-FDG a été pratiquée [18]. Dans notre série, 6 nouvelles localisations osseuses secondaires ont été mises en évidence grâce à la TEP. L'existence de faux positifs est liée aux processus inflammatoires et aux affections granulomateuses responsables de lésions pulmonaires nodulaires, dont les principales dans les séries européennes sont: lamycobactériose, la sarcoïdose, l'aspergillose et l'anthraco-silicose. Les séries américaines, quant à elles, citent essentiellement l'histoplasmose et la coccidioïdomycose [16]. Dans notre série, deux cas de faux positifs ont été relevés après biopsie par médiastinoscopie: l'un en faveur d'une anthracosilicose et le second en faveur d'une adénite réactionnelle. Le risque de faux négatifs est important dans la détection de nodules infra-centimétriques, surtout que la résolution des appareils TEP actuels se limite à 5 à 6mm [19]. Une autre cause de faux négatifs est liée au caractère hypo métabolique de certaines tumeurs particulières ne fixant pas le FDG tels que: les carcinomes «bronchiolo-alvéolaires», terme obsolète faisant référence à différentes entités: adénocarcinome in-situ, adénocarcinome micro-invasif, adénocarcinome invasif lépidique prédominant, adénocarcinome invasif mucineux. Les tumeurs carcinoïdes typiques. Certaines lésions kystiques à paroi fine. Un seul cas de faux-négatif a été relevé dans notre série, correspondant à une tumeur bronchiolo-alvéolaire. La TEP au FDG a par ailleurs un impact reconnu sur la détermination des volumes de référence en radiothérapie. Elle permet d'exclure du champ d'irradiation une simple atélectasie en aval de la tumeur ou d'y inclure des ganglions envahis que la TDM n'avait pas permis de déceler [**20**]. Deux patients de notre série ont bénéficié de la TEP-TDM pour déterminer la masse à irradier.

## Conclusion

Depuis son introduction en routine clinique, l'imagerie métabolique par TEP au 18 F-FDG a acquis une place bien établie dans la prise en charge des patients en oncologie thoracique en particulier dans le CBNPC. Elle intervient dans les différentes étapes du diagnostic, de la prise en charge et du suivi de ces patients.

### Etat des connaissances actuelles sur le sujet

- Le cancer broncho-pulmonaire (CBP) est le plus fréquent dans le monde et constitue le premier cancer chez l'homme;
- Il a un caractère généralement agressif qui peut rapidement engendrer des métastases et souvent diagnostiqué à un stade avancé;
- La TEP-TDM au 18F-FDG représente actuellement l'examen d'imagerie le mieux adapté pour évaluer l'extension initiale de ce type de cancer et peut modifier la prise en charge du cancer.

### Contribution de notre étude à la connaissance

- Cette étude montre la supériorité de la TEP-TDM en comparaison avec la TDM seule dans l'optimisation de la prise en charge du CBNPC, notamment dans le bilan d'extension locorégional et à distance ce qui concorde avec la littérature.

## Conflits d’intérêts

Les auteurs ne déclarent aucun conflit d'intérêts.
